# Acute hyponatremia after cardioplegia by histidine-tryptophane-ketoglutarate – a retrospective study

**DOI:** 10.1186/1749-8090-7-52

**Published:** 2012-06-10

**Authors:** Gregor Lindner, Bernhard Zapletal, Christoph Schwarz, Wilfried Wisser, Michael Hiesmayr, Andrea Lassnigg

**Affiliations:** 1 Department of Emergency Medicine, Inselspital University Hospital Bern, 3010, Bern, Switzerland; 2 Department of Cardiothoracic and Vascular Anesthesia and Intensive Care Medicine, Medical University of Vienna, Vienna, Austria; 3 Department of Nephrology, Medical University of Graz, Graz, Austria; 4 Department of Cardiac Surgery, Medical University of Vienna, Vienna, Austria

**Keywords:** Hyponatremia, Minimally invasive aortic valve replacement, Bretschneider cardioplegia

## Abstract

**Background:**

Hyponatremia is the most common electrolyte disorder in hospitalized patients and is known to be associated with increased mortality. The administration of antegrade single-shot, up to two liters, histidine-tryptophane-ketoglutarate (HTK) solution for adequate electromechanical cardiac arrest and myocardial preservation during minimally invasive aortic valve replacement (MIAVR) is a standard procedure. We aimed to determine the impact of HTK infusion on electrolyte and acid–base balance.

**Methods:**

In this retrospective analysis we reviewed data on patient characteristics, type of surgery, arterial blood gas analysis during surgery and intra-/postoperative laboratory results of patients receiving surgery for MIAVR at a large tertiary care university hospital.

**Results:**

A total of 25 patients were included in the study. All patients were normonatremic at start of surgery. All patients developed hyponatremia after administration of HTK solution with a significant drop of serum sodium of 15 mmol/L (p < 0.01). Measured osmolality did not change during all times of surgery compared to start of surgery (p = 0.28 – p = 0.79), indicating isotonic hyponatremia. After administration of HTK solution pH fell significantly due to development of metabolic acidosis.

**Conclusions:**

Acute hyponatremia during cardioplegia with HTK solution is isotonic and should probably not be corrected without presence of hypotonicity as confirmed by measurement of serum osmolality.

## Short summary

Hyponatremia is common after the administration of histidine-tryptophane-ketoglutarate (HTK) solution for induction of cardioplegia in cardiac surgery. However, the phenomenon is mentioned in only one recent study and there are no treatment recommendations for anesthetists. In our study, we show that the hyponatremia associated with the administration of HTK solution is iso-osmotic and should not be corrected in order to protect the patient from the serious consequences of hyperosmolarity.

## Background

Hyponatremia is defined as a serum sodium below 135 mmol/L and is associated with hypoosmolality in most cases [1]. Rapid development of hyponatremia can lead to cerebral swelling which can ultimately end in herniation and even death [1,2]. Thus, acute hyponatremia warrants acute treatment by raising the serum sodium level to avoid the development of brain edema. However, not only hyponatremia itself but its treatment may be harmful: The acute treatment of chronic hyponatremia, which is already at least partly compensated by several adaptation mechanisms (i.e. shift of osmotically active particles from the intra- to the extracellular space), can result in the probably most feared complication in the treatment of hyponatremia: central pontine myelinolysis [1,3,4]. Thus, thorough evaluation of the patient and the history of the development of hyponatremia are essential when rapid correction of hyponatremia seems to be indicated.

During open heart surgery, the administration of a cardioplegic solution for adequate electromechanical cardiac arrest and myocardial preservation is essential to perform the procedure. For minimally invasive aortic valve replacement (MIAVR) performed through a lateral right minithoracotomy or hemisternotomy, antegrade single-shot crystalloid histidine-tryptophan-ketoglutarate (HTK) cardioplegia is administered.

HTK cardioplegia is widely used to induce electromechanical cardiac arrest for several indications [5-8]. HTK solution contains only 15 mmol/L of sodium chloride and thus can be considered a hyponatremic solution. However, the osmolality of the solution is 310 mosmol/kg and is therefore slightly hypertonic. The composition of HTK solution is given in Table 1.

**Table 1:** Composition of HTK solution

	**mmol/L**
**Sodium chloride**	15
**Potassium chloride**	9
**Potassium hydrogen 2-ketoglutarate**	1
**Magnesium chloride 6 H**_**2**_**O**	4
**Histidine HCl H**_**2**_**O**	18
**Histidine**	180
**Tryptophan**	2
**Mannitol**	30
**Calcium chloride 2H**_**2**_**O**	0.015
**Osmolarity**	310
**pH at 25 °C (77 °F)**	7.02-7.2

Relative costs decrease from class 1 through class 7.

Since HTK solution is almost sodium-free, rapid infusion (within minutes) of up to two liters (or even more in some cases) might result in severe electrolyte derangements as described in a collective of pediatric cardiac patients [9]. Thus, in this retrospective analysis we aimed to investigate the effects of cardioplegia by HTK solution on sodium and acid–base state of patients receiving surgery for MIAVR.

## Methods

In this retrospective study, between July 2009 and February 2010 patients who received surgery for MIAVR whose data were available at the Department of Cardiac Surgery of the Medical University of Vienna were included in the study. Additionally, patients who received MIAVR during March 2011 were included, since for these patients serial measurements of serum osmolality during surgery were available.

Data were extracted from the automatic anesthesia reporting system and the prospective database of the Department of Cardiothoracic and Vascular Anesthesia and Intensive Care Medicine. Patient characteristics such as age, gender, height, weight, diagnosis and indication for operation were obtained. Data on operation time (cardiopulmonary bypass and aortic cross-clamp time), administered fluids, arterial blood gas analysis, which were taken approximately every 30 min, measured serum osmolalities (available for 7 patients) were gathered for the included patients.

All data were collected by the same person. Descriptive statistics, correlations and paired t-tests were calculated using Statistica 10, StatSoft Inc. Tulsa, Oklahoma. Data are given as median and first and third quartile when appropriate.

The study was approved by the Ethics Commission of the Medical University of Vienna.

## Results

Between July 2009 and February 2010 18 patients who received surgery for minimally invasive aortic valve replacement could be included into the study. Additionally, during March 2011, 7 patients with serial measurements of serum osmolality were available for inclusion into the study resulting in a total of 25 patients. Details on surgery are given in Table 2.

**Table 2:** Details on surgery. Characteristics are given in absolute numbers and percent or median and first and third quartile

**Indication for MIAVR**	
**- Aortic stenosis**	18 (72%)
**- Aortic insufficiency**	2 (8%)
**- Bicuspid aortic valves**	2 (8%)
**- Complex vitium**	2 (8%)
**- Combined aortic vitium**	1 (4%)
**Surgical access**	
**- Minithoracotomy**	18 (72%)
**- Hemisternotomy**	7 (28%)
**Aortic clamp time**	97 min (Q1: 75, Q3: 116)
**Extracorporeal time**	135 min (Q1: 116, Q3: 166)

Compression is higher for data that are easy or inexpensive to reproduce, and lower for data derived from unique or irreproducible samples.

13 (52%) patients were male. Median age was 67 years (Q1: 55, Q3: 74), median body height was 172 cm (Q1: 164, Q3: 174) and body weight was 86 kg (Q1: 75, Q3: 97). Total body water (TBW) as calculated by, TBW = weight * 0.5 (for women)/0.6 (for men), was 45 liters (Q1: 39.5, Q3: 58).

Patients received a median of 2000 ml (Q1: 2000, Q3: 2000) of HTK solution with a minimum of 1.000 ml and a maximum of 5.000 ml. Additionally, patients received 700 ml (Q1: 700, Q3: 900) of lactated Ringer’s and 1000 ml (Q1: 1000, Q3: 1000) of hydroxyethylstarch 130/0.4. A median of 570 ml (Q1: 490, Q3: 900) of packed red cells were administered during operation.

### Serum sodium during cardioplegia with HTK solution

**Figure 1:**
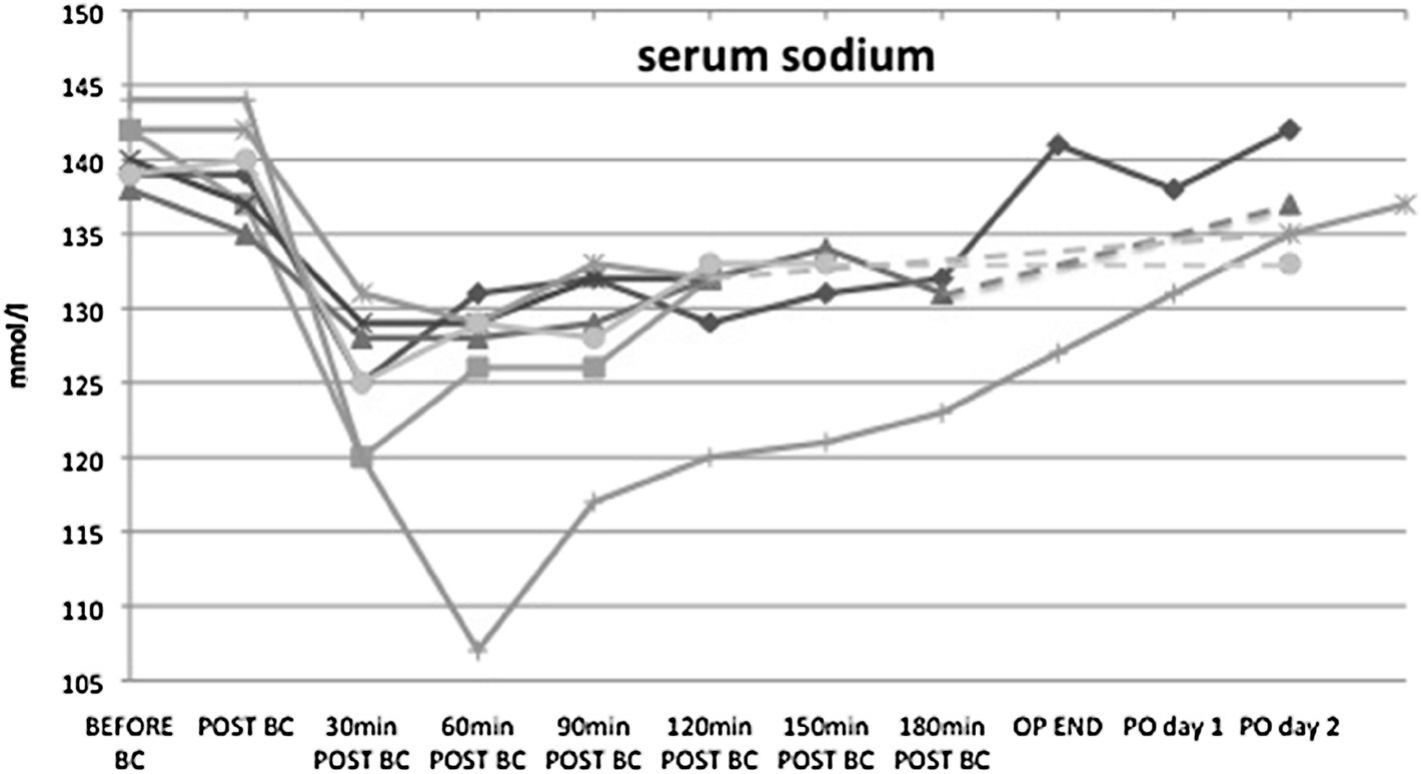
**Course of serum sodium of seven patients with measured serum osmolalities after administration of HTK.** While ABG stands for arterial blood gas analysis. Values in mmol/L.

Median serum sodium before start of cardiopulmonary bypass was 140 mmol/L (Q1: 139, Q3: 141). After administration of HTK solution for cardioplegia median serum sodium dropped to 124 mmol/L (Q1: 122, Q3: 126) with a minimum of 107 mmol/L in one patient. After application of HTK solution for cardioplegia median change in serum sodium concentration was 15 mmol/L (Q1: 13, Q3: 19) with a maximum of 37 mmol/L in one patient. Course of serum sodium for the seven patients with measured serum osmolalities is given in Figure 1.

Seven patients received between 25 and 100 ml 8.4% sodium-bicarbonate intraoperatively due to hyponatremia, no other hypertonic saline solutions (i.e. sodium-chloride 3%, 10% or 20%) were administered during anesthesia.

Serum sodium on postoperative day one, available for 21 patients, was 135 mmol/L (Q1: 133, Q3: 137), while serum sodium on postoperative day two and three was 137 mmol/L (Q1: 134, Q3: 138) and 137 mmol/L (Q1: 135, Q3: 138), respectively.

Calculated effective osmolality for all patients computed as:

(1) $${\text{Effective Osmolality}}_{\text{calculated}}=2x\left[{\text{Na}}^{+}\right]+\left[\text{Glucose}\left(\text{mmol}/\text{L}\right)\right]$$

was 286 mmol/L (Q1: 283, Q3: 289) before and 254 mmol/L (Q1: 250; Q3: 257) after the administration of HTK solution (p < 0.01).

**Figure 2:**
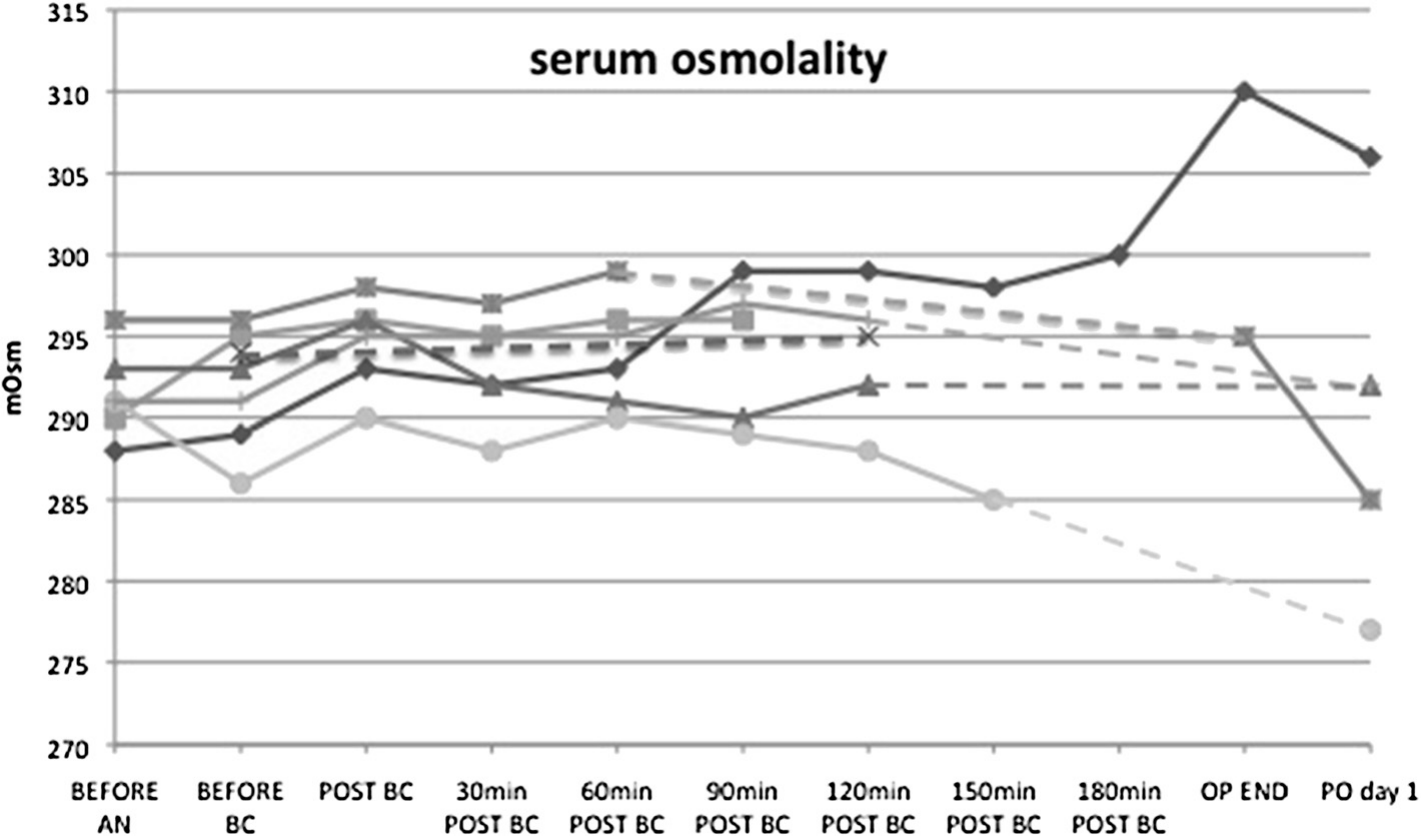
**Course of measured serum osmolality of seven patients with available serial measurements of serum osmolality before and after administration of HTK.** Values in mmol/L.

For all patients included during March 2011 serial measurements of serum osmolality were available. Serum osmolality before start of extracorporeal time was 293 mmol/L (Q1: 289, Q3: 295). After administration of HTK solution serum osmolality was 294 mmol/L (Q1: 292, Q3: 295). During no time of surgery, serum osmolality changed significantly compared to the serum osmolality before the administration of HTK solution (p = 0.28 – p = 0.79). The course of serum osmolality before and after administration of HTK solution is given in Figure 2. The one patient who received 8.4% sodium-bicarbonate showed the highest change in measured serum osmolality: It rose from 288 mmol/L at start of operation to 310 mmol/L at the end of surgery.

### Acid–base state during cardioplegia with HTK solution

Before start of cardiopulmonary bypass and administration of HTK solution for cardioplegia, pH was 7.419 (Q1: 7.4, Q3: 7.434), partial pressure of carbon dioxide was 38 mmHg (Q1: 35, Q3: 40), bicarbonate was 25 mmol/L (Q1: 24, Q3: 26) and base excess was 0.3 (Q1: -0.6, Q3: -1.5). Serum lactate concentration was 1.1 (Q1: 0.7, Q3: 1.4).

After administration of HTK solution for cardioplegia pH decreased to 7.32 (Q1: 7.27; Q3: 7.34) (p < 0.01) and bicarbonate to 19 mmol/L (Q1: 18, Q3: 20) (p < 0.01). Partial pressure of carbon dioxide was 39 mmHg (Q1:37, Q3: 39) (p = 0.2). Base excess decreased to −6 (Q1: -7, Q3: -5) (p < 0.01). Serum lactate concentration rose to 1.5 mmol/L (Q1: 1.4, Q3: 1.8) (p < 0.01). None of the patients showed an increased plasma anion gap before or after the administration of HTK.

No intraoperative deaths were noted, post operative monitoring of all patients was routinely conducted in the ICU and no evidence of any neurological abnormalities was found in any patient during that period. 92% (23) of all Patients could be relesased from the ICU alive, 8% (2 patients) died due to multi organ failure.

## Discussion

In this study we present 25 patients receiving surgery for MIAVR. All of the patients experienced significant drops of serum sodium of 15 mmol/L after administration of HTK solution. However, no change in measured osmolality occurred after the decrease in serum sodium. Thus, although patients experienced massive declines in serum sodium concentration, no change in osmolality was observed. Metabolic acidosis occurred in all patients after administration of HTK solution.

Recently, Kim and coworkers published a study on the effect of two different cardioplegic solutions on serum sodium in a pediatric collective of cardiac patients [9]. In their study, they found that use of HTK solution was significantly associated with the occurrence of hyponatremia. However, measured serum osmolalities were not reported. An overall postoperative seizure incidence of 2.9% in this collective with a median age of 6 months might not be causally related to the presence of hyponatremia. Usually, acute development of hyponatremia (e.g. after infusion of 5% dextrose solution) is a life threatening condition [1,10]. During hypotonic hyponatremia a shift of free water from the extracellular to the intracellular space happens, resulting in cellular swelling [2]. This causes problems especially in the brain, when cerebral edema can lead to herniation or even death [1]. However, this only happens in hypotonic hyponatremia. In the patients presented here, serum osmolality remained constant during the whole time of surgery. Although HTK solution contains only 15 mmol/L of sodium, its osmolality is 310 mmol/L making it a slightly hypertonic solution. This is because its osmolality is mainly constituted by amino acids. A comparison of the Edelman equation and the constitution of osmolality immediately explains why the hyponatremia, which occurs after administration of HTK solution is of the isoosmotic type. The Edelman equation depicts the serum sodium as a function of total exchangeable sodium and potassium and total body water [11]:

(2) $$\left[{\text{Na}}^{+}\right]={\left({\text{Na}}^{+}+{\text{K}}^{+}\right)}_{\text{exchangeable}}/\text{TBW}\text{.}$$

While (Na^+^ + K^+^)_exchangeable_ stands for the amount of total exchangeable sodium and potassium in the body and TBW for total body water. According to the Edelman equation, hyponatremia can only develop when either there is a loss of sodium (or potassium) on the one hand or a gain of water, which augments the denominator of the formula, as it is the case in administration of the (almost) sodium free HTK solution. However, this has to be distinguished from the constitution of serum osmolality which is defined by [2]:

(3) $${\text{Osmolality}}_{\text{Serum}}=\text{Total body osmoles}/\text{TBW}\text{.}$$

When administering one liter of HTK solution with an omsolality of 310 mmol/L, which is only slightly hypertonic to plasma, one has to add the 310 mmol/L to the enumerator, and the 1 liter of volume to the denominator. In fact, the osmolality doesn’t change or at most will slightly increase as was seen in our patients.

Thus, the administration of HTK solution to a patient results in isotonic hyponatremia. The danger in this situation lies in a misinterpretation of laboratory results: when only serum sodium concentration is available (as it is often the case with only arterial/venous blood gas analysis) the rapid decline in serum sodium after administration of HTK solution for cardioplegia might provoke administration of hypertonic saline by the anesthetist in order to correct for acute hyponatremia. This in turn would result in hypertonicity and consequences comparable to acute hypernatremia. Therefore, no correction should be initiated without measured serum osmolality. In our study, metabolic acidosis developed after the administration of HTK solution. Although serum lactate concentration rose significantly, its absolute rise was negligible. The development of metabolic acidosis under circumstances of cardioplegia with HTK solution can most likely be explained by the mechanism of dilution acidosis [2]: The rapidly administered bicarbonate free HTK solution (approximately two liters in our patients) leads to rapid volume expansion and consequent dilution of bicarbonate and metabolic acidosis. However, the significance of these findings is not clear.

Our study is limited by its retrospective design and lack on long-term outcome. Measured serum osmolalities were only available for 7 patients. Measurements of amino acid concentration after administration of HTK solution were not available for any of the patients included in this study.

## Conclusions

The administration of HTK solution in patients receiving surgery for MIAVR results in marked declines in serum sodium. However, serum osmolality remained stable during surgery indicating presence of isotonic hyponatremia not requiring treatment. In fact correction of hyponatremia during/after cardioplegia with HTK solution might cause hypertonicity with its associated adverse events.

## Competing interests

The authors declare that they have no competing interests.

## Authors’ contributions

GL was involved in the conception of the study, performed the analysis and interpretation of the data, wrote the article and gave his final approval to the article. BZ gathered data for the study, helped in drafting the manuscript and gave his final approval to the article. CS performed significant work in the analysis and interpretation of data, helped in drafting the manuscript and gave his final approval to the article. WW was involved in the conception of the study, helped in drafting the manuscript and gave his final approval to the article. MH was involved in the conception of the study, helped in drafting the manuscript and gave his final approval to the article. AL was involved in the conception of the study, gathered data and performed significant work in the analysis and interpretation of data, helped in drafting the manuscript and gave her final approval to the article. All authors read and approved the final manuscript.
